# Dialogic Priming and Dynamic Resonance in Autism: Creativity Competing with Engagement in Chinese Children with ASD

**DOI:** 10.1007/s10803-022-05505-2

**Published:** 2022-03-31

**Authors:** Vittorio Tantucci, Aiqing Wang

**Affiliations:** 1 Department of Linguistics and English Language Lancaster University, Lancaster, UK; 2 Chinese Department of Modern Languages and Cultures, University of Liverpool, Liverpool, UK

**Keywords:** Pragmatics, Priming, Resonance, Mandarin, Corpus linguistics, Creativity

## Abstract

A growing body of research has focused on the relationship between priming and engagement through dialogue (e.g. Tantucci and Wang in Appl Linguist 43(1):115–146, 2022; Mikulincer et al. in Cognit Emotion 25:519–531, 2011). The present study addresses this issue also in relation to creativity and provides a new applied model to measure intersubjective engagement in ASD vs neurotypical populations’ speech. We compared two balanced corpora of naturalistic Mandarin interaction of typically developing children and children diagnosed with ASD (cf. Zhou and Zhang in Xueqian jiaoyu yanjiu [Stud Preschool Educ] 6:72–84, 2020). We fitted a mixed effects linear regression showing that, in both neurotypical and ASD populations, dialogic priming significantly correlates with engagement and with whether the child could creatively re-use the original input to produce a new construction. What we found is that creativity and intersubjective engagement are in competition in children with ASD in contrast with the neurotypical population. This finding points to a relatively impeded ability in ASD to re-combine creatively a priming input during the here-and-now of a dialogic event.

## Introduction

The present study is centered on morphosyntactic and pragmatic creativity as a by-product of engagement in children with Autism spectrum disorder (ASD). We designed a corpus-based model to assess whether children with ASD would engage with the language of a peer via mere repetition or whether they would re-combine the utterance they heard in order to express something new and thus engage creatively throughout an ongoing interaction.

To achieve this, we retrieved 2000 utterances that were characterised by syntactical or lexical analogy from a preceding turn at talk from the corpora of first language acquisition of Mandarin Chinese Zhou2, Zhou3 (Li & Zhou, 2004; Zhang & Jing, 2009) and the Shanghai corpus of children with ASD (cf. Zhou & Zhang, 2020). We developed an annotation model accounting quantitatively for the degree of syntactical and lexical similarity and creative variation of the child utterance in relation to a previous utterance through dialogue, within a distance of three turns at talk. In the framework of Dialogic syntax (cf. Tantucci and Wang, 2021, 2022; Du Bois, 2014) the re-elaboration of a dialogic prime^1^ at talk involves resonance, which has to do with the ability to re-use a linguistic form that has been encountered through an interaction (cf. Tantucci and Wang, 2021, 2022; Du Bois, 2014). Our analysis indicates that children with ASD displayed a comparatively more impeded capacity to resonate creatively—rather than via mere repetition—with the utterances that they encountered through a dialogue. Furthermore, they showed a more inhibited ability to resonate creatively with a preceding linguistic stimulus in combination with sentence final particles of intersubjective engagement (SFP), which in Mandarin Chinese constitute a non-obligatory grammaticalised category (Tantucci, 2021; Tantucci and Wang, 2018, 2020a, b). Finally, we found a higher degree of morpho-syntactic creativity when children with ASD resonate with their own linguistic utterances, in the form of self-priming, as intersubjective engagement had a comparatively weaker effect on their ability to re-use creatively a dialogic input.

The present study advances the theory and the methods of usage-based research on ASD, as it provides an applied model to measure the degree of formal engagement of the child with what s/he just heard. More importantly, this novel framework allows the analyst to identify on a large scale the degree to which the child makes an overt effort to re-elaborate the dialogic prime (i.e. an utterance produced in a dialogue) that s/he has encountered in order to express something new. This entails the possibility to measure engagement against creativity and to evaluate whether—and how—these two dimensions correlate in typically developing (TYP) vs ASD naturalistic speech. Finally, this approach also relies on the intersubjective gradience model (Tantucci, 2021), whereby intersubjective awareness is linguistically expressed in the form of an extra-propositional surplus of meaning that is additional to the goal-oriented dimension of an utterance. The intersubjective dimension is highly grammaticalised at sentence periphery in languages of the South East in the form of non-obligatory sentence final particles (SFP). SFP are used by interlocutors to overtly express their awareness of the addressee’s potential reactions to what is being said. For instance, an assertion such as *London is very beautiful*, in Mandarin may include the SFP 吧 *ba* (cf. Tantucci, 2017b) to mark intersubjectively the expectation that hearer will agree with what is being said (as in 伦敦 *lúndūn* ‘London’ 挺 *tǐng* ‘very’ 漂亮 *piàoliang* ‘beautiful’ 吧 *ba*). In this sense, Mandarin system of SFPs represent a precious resource for the usage-based and naturalistic enquiry of intersubjectivity.

This study examined four primary research questions:

1. Is the degree of resonance the same in 42-to-60-months-old TYP children compared with children with ASD?
2. Is there a difference across the two populations concerning whether resonance occurs statically (as mere repetition) rather than creatively (as a re-elaboration of a dialogic prime)?
3. What is the relationship (if any) between resonance and overt usage of sentence final particles of intersubjective engagement (SFP) in TYP children and ones with ASD?
4. Finally, what is the relationship between resonance and the source of a prime, viz. whether a dialogic stimulus originates from the mother or the child him/herself?

The paper first reviews the experimental and the linguistics’ literature on Autism and interactional engagement. In the following section, we introduce the notions of resonance and intersubjectivity and their relationship with the internal constituency of a construction as it has been theorised in Dialogic Syntax. We then discuss the data retrieval, the annotation model and the statistical analysis of our study. In particular, we provide the results from a multifactorial mixed effects linear regression and a conditional inference tree model, both aimed at measuring the degree and the modality of syntactic resonance in the two populations of this study. We then discuss the results of our analysis and provide novel insights about ASD, engagement and creativity. Finally, we formulate the conclusions of our study.

## Autism, Implied Meaning and Idiomatic Language

Autistic spectrum disorder (ASD) is often diagnosed with reference to difficulties in the use of language and communication for social purposes (American Psychiatric Association, 2013; World Health Organisation, 2018). It is yet attested that around 70% of the individuals on the autistic spectrum do eventually reach functional language (Anderson et al., 2007; Kim et al. 2014; Wodka et al., 2013). Interactional meaning is often conveyed indirectly or metaphorically. In this respect, Deliens et al. (2018) discuss ASD individuals’ ability to generate indirect request interpretations and comprehension of irony. They propose that ASD individuals are relatively geared towards an egocentric processing of context and struggle to make assumptions about the interlocutor’s mental states. Similar difficulties are often reported when the communicated content does not correspond to the literal linguistic interpretation of the utterance (Tantucci, 2021). This involves problems in comprehending metaphors, idioms, conversational inferences, indirect speech acts, jokes and irony (e.g. Happé, 1993; Loukusa et al., 2007; MacKay & Shaw, 2004; Martin & McDonald, 2004; Ozonoff & Miller, 1996; Paul & Cohen, 1985; Surian, 1996). Impeded idiomatic thinking has been linked to impeded ability of making assumptions about other people’s mental states (e.g., Baron-Cohen, 1992, 2000; Happé, 1995; Heavey et al., 2000; Joliffe & Baron-Cohen, 1999; Senju et al., 2010; Yirmiya et al., 1998). Nonetheless, such a ‘uniform pragmatic impairment’ view, has also been challenged both on theoretical and empirical grounds (e.g. Brock et al., 2008; Hermann et al., 2013; Norbury, 2005). Deliens et al (2018) note that ASD pragmatic impairment may be thus a matter of degree—or quality—rather than an absolute deficiency. In this regard, Kissine (2012, 2013, 2016) proposes to distinguish between shallower pragmatic processes that draw on contextual factors to select between several available interpretations, but do not require adopting one’s conversational partner’s perspective, and those that are rooted in complex mind-reading abilities. In Tantucci’s intersubjective gradience account (2020, 2021), interactional co-operation ranges from ego-centric engagement, to overt mentalising abilities involving the awareness of a specific interlocutor’s mind (immediate intersubjectivity) and—at a higher degree of complexity—the awareness of how anyone in society may react to what is being currently said (extended intersubjectivity). In the present study, overt marking of intersubjectivity will appear to be in competition with creativity in ASD speech.

## Autism and Dialogic Engagement

While many individuals with ASD develop semantic language skills which can be compared to the typically developing (TYP) population, nonetheless ASD individuals demonstrate difficulties in pragmatic abilities, which inhibit engagement in reciprocal conversations and social interactions (Eigsti et al., 2011; Howlin et al., 2013; Knott et al., 2006; Volden, 2017). For instance, research on friendship interactions indicates lower ratings of conversational flow in ASD vs TYP populations (Bauminger et al., 2008). Interaction of individuals with ASD has been argued to markedly include lack of eye contact (Ames & Jarrold, 2007; Hobson & Lee, 1998; Pisula, 2010; Tager-Flusberg, 1999; Wiklund, 2016) and echolalia (Sterponi & de Kirby, 2016). People diagnosed with ASD have also shown a tendency to struggle to adapt to common ground and pragmatic context (i.e. Gernsbacher et al., 2005; Lord & Paul, 1997; Tager-Flusberg, 2000; Tager-Flusberg et al., 2005). Scarce use of referential expressions, difficulties in constructing a coherent narrative, avoiding redundant messages and poor tuning into the conversational flow have been attested in autistic children and adults (e.g. Asp & de Villiers, 2010; Baixauli et al., 2016; Baltaxe & D’Angiola, 1992; Colle et al., 2008; Diehl et al., 2008; Eales, 1993; Fine et al., 1994; Surian et al., 2007). Other pragmatic difficulties that have been reported in individuals with ASD, include initiating conversation, repairing misunderstandings, perseverating on topics, and making topically relevant comments (Kissine, 2012; Loveland et al., 1988; Volden, 2017). Based on recorded conversations of 46 participants with autism or Asperger syndrome, De Villiers et al. (2007) identified monotonous speech, abrupt topic shifts, low rates of initiations and short responses, topic perseveration, proffering of information that is not commensurate with what is required, repetitions or self-corrections, echolalic or self-stimming noises and an inability to stay on topic.

Direct observations of infants (e.g. Charman et al., 1997) and parental reports (e.g. Wimpory et al., 2000) show evidence of young children with autism having impairments in non-verbal communication that could reflect disruption in intersubjective engagement. From the angle of Conversation Analysis, Ochs et al. (2005) and Dobbinson et al. (1998) noted that people with ASD have a marked tendency to respond to interlocutors’ turn-taking by adjusting to the immediate, but not the global topic of discourse. Similarly, difficulties have been reported in conforming to conversational rules such as initiating and engaging in reciprocal conversations (Ball, 1978; Baltaxe & Simmons, 1977; Fine et al., 1994). There is evidence that such deficits may be connected to the child’s broader social and communicative impairments, for example as assessed by the Autism Diagnostic Observation Schedule (Eales, 1993; Hale & Tager-Flusberg, 2005; Lord & Paul, 1997; Tager-Flusberg, 2000) and as reflected by a comparatively reduced ability to engage in shared attention (Rollins & Snow, 1998).

A crucial issue in the literature of ASD speech is that interactional mismatches between people with ASD and TYP have often been confined to a lab and conducted under artificial clinical or experimental conditions (Apperly, 2010; Tantucci, 2020, 2021; Sng et al., 2020). It is no secret that the current state of art of linguistic research on ASD critically needs to be implemented by results originating from large-scale, lab-free, naturally occurring conversation. Individuals with ASD tend to fare better in structured and predictable interactions involving familiar people and familiar adults in particular (Lord & Magill, 1989). This suggests that, in naturalistic speech, recurrent interactional cues may be preferred to ones that include a component of novelty. This is a crucial aspect of the present study, as we focus on individuals’ ability to re-use a prime in a novel way as a byproduct of engagement. In fact, while it has been attested that, to some degree, children with ASD are able to learn new semantic information in context (Lucas et al., 2017), it is yet to be determined whether and to what degree they can produce novel constructional structures out of a prime in naturalistic conversation and creatively inhibit repetitive behaviours (cf. Tantucci and Di Cristofaro, 2020).

## Resonance and Intersubjectivity

From a usage-based perspective, human beings’ subjective experiences are coordinated with the experiences of others (Hobson, 1993, 2002; Tomasello, 1999). Nonetheless, Cognitive linguistics have been originally centered on individual ability to conceptualise things and processes (e.g. Langacker, 1987, 1991; Talmy, 2000), involving the holistic processing of form and meaning (Croft & Cruise, 2004). Over the last two decades, linguistic constructions (e.g. Fillmore, 1988; Golderg 1995, 2006) have yet been finally approached as a byproduct of dialogic interaction. Language has thus been studied as a joint activity (cf. Clark, 1996), as utterances are no more viewed as independent monads of form and meaning, but rather as interactional tools that are dynamically reviewed and recalibrated across turns at talk (Dingemanse, 2020, p. 24). The emergence of grammar (Thompson & Hopper, 2001) and constructional creativity (Tantucci and Wang, 2021, 2022) from naturalistic interaction is one of the tenets of Dialogic Syntax (cf. Tantucci and Wang 2018; Du Bois, 2014; Zima & Brône, 2015). In this view, constructions result from interlocutors’ dialogic engagement, with analogies constantly being realised at phonological, semantic-pragmatic, and syntactic levels (Du Bois & Giora, 2014; Du Bois, 2014).

A fundamental dimension for the analysis of intersubjective engagement in naturalistic interaction is **resonance**, which is realised in the form of analogies across utterances and constructions through dialogue. The key aspect of resonance is that it is a formal indicator of interactional engagement, as one speaker draws on a prior utterance as a resource for producing a new one and selectively re-uses some of the words, structures, and other linguistic resources that were just uttered . As an example, consider the following exchange:

**Table:** 

(1)	A: What do you like most about yourself David?	
	B: I like my nose, my nice clothes	
		(Du Bois, 2014, p. 417)

In the excerpt above, the child ‘B’ picks up on the interviewer’s chunk *you like* and produces the corresponding construct *I like*, with verbatim reproduction (*like*: *like*) and the substitution of first person for second person pronoun. On the one hand, the child retrospectively engages with the prior speaker’s contribution through ad hoc analogies with the form and meaning of the earlier utterance. On the other hand, a verbal framework for what is to come is also established, as given information (*I like*) is to support the child’s introduction of new information (*my nose, my nice clothes*) (cf. Du Bois, 2014, p. 417). Analogies from one construction to another have implications for verbal scaffolding (Vygotsky, 1986; Wertsch, 1998), dialogic affordance (Gibson, 1979) and for structural/constructional priming (Allen et al., 1997; Tantucci & Wang, 2021, 2022; Bock, 1982; Garrod & Pickering, 2004; Gries, 2005; Pickering & Garrod, 2004). As shown in Du Bois and Giora (2014),  and Tantucci and Wang (2021, 2022) resonance pervades the organisation of turn-taking sequences in naturalistic interaction. In this respect, Du Bois (2014) notes that a systematic analysis of resonance can reveal something that has proved elusive in ASD individuals’ abilities to achieve intersubjectively attuned communication with others.

## Resonance and Creativity

In some cases, resonance merely leads to simple replication of a previous linguistic input, while in some others it involves creativity. When resonating constructs are formally and functionally equivalent with the input, imitation is clearly less complex and mere repetition is at play. This often occurs in language acquisition, whereby children copy a specific priming input as a learning process, without any creative intervention. The present paper will refer to these instances as cases of **static resonance**. See for instance example (2) below:

**Table:** 

(2)		
MOT:	这两个是小松树。	
	zhè liǎng ge shì xiǎo sōngshù	
	this two CLASS be little pine tree	
	‘These are two little pine trees.’	
MOT:	表现好不好。	
	biǎoxiàn hǎo bù hǎo	
	behave goo not goog	
	‘Behave, come on.’	
CHI:	小松树。	
	xiǎo sōngshù	
	little pine tree	
	‘Little pine trees.’	
		CHILDES / Shanghai / Wang / 48

The case above does not involve creativity. The noun phrase 小松树 *xiǎo sōngshù* ‘small pine’ uttered by the mother (MOT), is subsequently re-uttered by the child without any overt morphosyntactic or functional contribution to the on-going conversation.

While cases such as (2) are the norm at early stages of ontogeny, nonetheless resonating constructions expectedly involve creativity along with the child’s development. These are instances in which the resonating construction includes the re-elaboration of the previous dialogic input. These are constructions involving dynamic resonance, as they have to do with the alteration of a linguistic form on the fly in ways that are meant to be comprehensible to those who were present in the dialogic moment (cf. Du Bois, 2014, p. 353). Pragmatically, dynamic resonance may underpin boosting, mitigating or reverting the illocutionary force of a preceding utterance (i.e. Tantucci, Culpeper and Di Cristofaro, 2018; Veale et al., 2006). In fact, children often use repetition strategically and creatively to achieve interactional goals (Corsaro & Maynard, 1996; de León, 2007; Ervin-Tripp, 1991; Goodwin, 1990, 2006; Keenan, 1977). To ease the interpretation of our results, in the present paper we will specifically refer to instances of resonance occurring dynamically as cases of **creative resonance**. The case below is from our dataset and involves a mother and a child facing a light:

**Table:** 

(3)		
MOT:	哦, 红灯啦!	
	O, hóng dēng la	
	Oh, red light LA	
	‘Oh, that’s a red light isn’t it!’	
MOT:	右手指出来嘛!	
	yòu shǒuzhǐ chūlái ma	
	Point it out with your right hand!	
CHI:	红灯停。	
	hóngdēng tíng	
	With a red light we must stop	CHILDES / Shanghai / Tianjie / 48

The creative analogy constructed by the child (CHI) after the mother’s (MOT) utterance can be represented in the form of a diagraph, viz. a constructional structure that emerges through the mapping of a structured relations among two or more utterances through dialogue (Du Bois & Giora, 2014, p. 354). The diagraph of the exchange in (3) is reported in Table 1, where creative alteration of the original utterance is marked as underlined text (in case of replacement) and in brackets (in case of (addition)).

**Table 1:** Diagraph of the emerging construction [Adj N SFP!]

	Adj	N	SFP!
MOT	*红*	*灯*	*啦*
CHI	*红*	*灯* (停)	*/*

*Adj* adjective, *N* noun

As the diagraph above shows, a priming attributive construction [Adj N SFP!] is uttered by MOT to warn CHI about a red light on the street. The construction carries both representative and directive illocutionary force, as the statement is uttered to inhibit the child to further proceed. The intersubjective sentence final particle (SFP) 啦 *la* is also added as a surplus of meaning emphasising the warning component of the utterance, together with the expected cooperation from CHI. In the subsequent turn at talk, CHI re-uses the linguistic material she has been primed by and formulates a new directive construction [红 *hóng* ‘red’ 灯 *dēng* ‘light’ 停 *tíng* ‘stop’], entailing collective intentionality (Tomasello, 2019) and extended intersubjectivity (Tantucci 2017a; Tantucci & Wang, 2020b; Tantucci, 2021) as to convey what people ought to do in those contextual conditions. Different from (2), in this case the child does not simply repeat a priming construction. Rather, creativity is now in action, as s/he re-elaborates what s/he just heard both formally and functionally in order to express something new.

## Engagement and Creativity in ASD Interaction

This is not the first account of resonance in ASD speech. In Hobson et al. (2012), children with ASD tended to produce instances of dialogic resonance that were often characterised by incoherent, truncated, vague, partly echoic, or non-responsive elaboration. Their results point to a close relation between impairments in intersubjectivity and a more impeded elaboration of dialogic discourse among individuals with autism. Hobson et al.’s study tackled whether children were able to express ‘grammatically correct’ sentences and discursive coherence in the form of dialogic adaptations. In the present work we are interested in the children’s ability to produce novel constructions after a dialogic prime throughout naturalistic (i.e. non-elicited) conversation. This means that our focus is on the correlation between engagement and creativity, rather than proficiency. Both Hobson et al. (2012) and Kissine (2021) suggest that dialogic engagement is partly—rather than entirely—impeded in children with ASD, emerging as a weaker, rather than missing, propensity to identify with the attitudes and stances-in-speaking of other people. We will tackle this hypothesis with an applied model of analysis of ASD corpus data to measure the degree of engagement and dialogic creativity that are involved in dialogic priming and complex imitation (cf. Arbib, 2012).

While priming has been often argued to occur as a distinctively structural and implicit mechanism (e.g. Bock 1986; Bock et al., 2007; Hurley, 2008), however, creativity emerged as an important dimension of priming in a study by Allen et al. (1997). The latter involved a game setting in which an experimenter and a child took turns in describing different pictures of unrelated content. The experimenter’s description was based on either an active or a passive construction. As predicted, the child’s subsequent description of a different picture showed increased use of the structure just heard. Their results showed that children with ASD were similar to typically developing children (matched for chronological or verbal age) in their capacity towards syntactic alignment. What was also found was that children both from TYP and ASD populations not merely repeated words verbatim, rather, they often re-used an abstract syntactic frame but with distinct lexical content to express a new message, i.e. what the present framework addresses as creative (dynamic) resonance. Du Bois et al. (2014) noted that in their own study participants with autism were often able to pick up some kind of linguistic “frame” from the interviewer, however there was often failure to assimilate this to their own stance in order to provide a coherent expansion of their own. While Du Bois et al. (2014) were mainly interested in stance coherence as a by-product of engagement, in the present study we focus on the way children with ASD are able to engage with a dialogic prime by adding an element of—morphosyntactic and/or functional—novelty to a dialogic prime, therefore being able to boost the conversation flow. Du Bois et al. (2014) remarked that “inevitably, [their] experimental approach involves some loss of subtlety when compared with the close analysis of naturally occurring conversation” (Du Bois et al., 2014, p. 430). In this sense, this paper aims to answer the call to look at how dialogic resonance works in everyday life, where conditions are more dynamic and less predictable, with richer social and affective structures (cf. Sterponi, 2004).

## Data Retrieval and Analysis

The data from TYP children were retrieved from the Zhou3 (cf. Zhang & Zhou, 2009) and Zhou2 (cf. Li & Zhou, 2004) corpora of first language acquisition, both comprising naturalistic interaction among children, peers and caregivers. We controlled for interactions that exclusively included children talking with their mother. The age-span of the Zhou3 corpus ranges from 0;08 to 4;05, while the Zhou2 corpus comprises an age-span between 3;05 and 5. We normalised the data of the two corpora so that the Zhou3 corpus would not exceed 37 months, with children’s turns therefore amounting to a total of 6143. We then randomly retrieved the same number of utterances from the Zhou2 corpus, with an overall TYP corpus comprising 12,286 utterances. We opted for utterances—rather than words—with the aim of capturing children’s progressive ability to formulate longer turn takings as they grow older. This may correlate with the hypothesis that children would develop increasingly sophisticated ability to resonate creatively rather than statically. The Shanghai corpus of ASD speech comprises interactions with children speaking with their mother raging from 37 to 56 months of age, with a total of 17,686 children’s turns at talk. The interactional setting among children and their mothers are comparable across the Zhou3, the Zhou2 and the Shanghai corpora as taking place in the children’s kindergarten, classrooms and home settings (cf. Zhou & Zhang, 2020).^2^

We selected the first 500 cases of resonance—either occurring statically or creatively—from children who were included in both the TYP and the ASD corpora respectively, ranging from 48 to 54 months of age. We then also retrieved the first 500 occurring instances of resonance from both corpora for children ranging from 55 up to 60 months old. Our dataset therefore comprised 2000 utterances overall, a half of which were spontaneously produced by TYP children and the other half by children with ASD. This retrieval method aimed to capture how resonance varies from one population to another, as it is already evidenced that resonance is indeed present in ASD populations to a similar extent as it is in TYP ones (e.g. Du Bois et al., 2014). In particular, we aimed to measure the degree of resonance across the two populations, that is, how much linguistic—either lexical or schematic—information is re-used by ASD children in contrast with TYP children. More importantly, we were interested in assessing the relationship between resonance and creativity in the two populations, hinging on whether the child dynamically re-uses a dialogic prime to express something new. Finally, we wanted to tackle the relationship between resonance and sentence peripheral marking of intersubjectivity. This allowed us to shed light on whether—and to what extent—intersubjective engagement is in competition with creativity in ASD and TYP populations.

## Annotation and Methodology

Our annotation was centered on the presence of resonance, viz. the overt repetition or reformulation of a lexical item, an interjection or a—more or less schematic—construction from a preceding turn at talk. A multifactorial scheme of annotation was developed (cf. Tantucci & Wang, 2021, 2022), which included the age of the child (the number of months), whether the utterance was marked intersubjectively via sentence final particles (SFP), the source of resonance (i.e. having to do with the child resonating with his/her interlocutor, with him/herself or with both), whether resonance occurred creatively or statically, the degree of lexical resonance and the degree of syntactic resonance. A sample line of the input of all these dimensions is given in Table 2.

**Table 2:** Sample of annotation

Months	SFP	Source	Res type	Lex Resonance	Synt Resonance
*48*	*la*	*other*	*creative*	*2*	*3*

The distinction between static and creative resonance hinges on whether the child simply repeated the mother’s dialogic prime or whether s/he would creatively re-use part of the priming construction in order to express something new. Lexical resonance was measured as a continuous variable by counting the number of words or interjections that were re-used by the child after a priming construction. In contrast, creative resonance involved the internal constituency of resonating ad hoc constructions. This means that syntactic resonance did not simply coincide with mere repetition of words, but rather with the number of internal constituents of schematic constructions that shared some common features with a preceding utterance. For instance, a priming construction [*I am so happy today*] would entail creative resonance in the form of [*I am also really happy today*], as they are both specific instantiations of the more schematic [Subj Copula INT^3^*happy today*] construction. The latter includes 5 components, hence the emerging value of syntactic resonance is 5. Distance from the prime to the resonating construct was limited to three turns at talk. We can look at example (4) below as an illustration of this annotation method:

**Table:** 

(4)		
MOT:	狐狸吓得逃跑了。	
	húli xià de táopǎo le	
	fox scared DE run-away LE	
	‘The fox was so sacred that it ran away.’	
MOT:	好了。	
	hǎo le	
	good LE	
	‘Good.’	
CHI:	吓得赶紧跑了。	
	xià de gǎnjǐn pǎo le	
	scared DE hurriedly run-away LE	
	‘So scared that it hurriedly ran.’	

In the case above, the mother (MOT) makes use of a complement of degree—also called resultative or V-DE construction (e.g. Chao, 1968; Dai, 1992; Li & Thompson, 1981)—which is introduced by the post-verbal particle 得 *de*, specifically expressing the outcome of some event. Literally, the mother subjectively describes the degree to which *the fox was scared* via the complementing clause 逃跑 *táopǎo* ‘run away’. The child then resonates with the construct via ellipsis of the subject and the addition of the adverbial 赶紧 *gǎnjǐn* ‘hurriedly’ and the reduction of the complementing verb in the monosyllabic form 跑 *pǎo* ‘run’. The key of this process is that the child creatively intervenes on the construction that s/he just heard and provides additional functional and morphosyntactic information, further elaborating on how *the fox ran away*.

Based on our scheme, the occurrence was annotated as involving the presence of the sentence final particle (SFP) 了 *le*. South East Asian languages are often characterised by a grammaticalised system of non-obligatory SFP, which are used to preemptively address the hearer's potential reactions to what is being said (cf. Tantucci and Di Cristofaro, 2021 on intersubjectivity and preemptive interaction). Mandarin has a sophisticated SFP system and represents a precious resource for the usage-based analysis of intersubjectivity counting as extra-propositional surplus of meaning throughout naturalistic dialogic exchanges (e.g. Tantucci, 2021; Chor, 2013; Haselow, 2012). In fact, evidence shows that children tend to develop the capacity to express immediate and further extended intersubjective functions of SFPs around their fourth year of age (Tantucci, 2021; Tantucci and Wang, 2020a). Most interestingly, this is roughly the same stage of ontogeny when mindreading abilities are argued to allow children to pass false-belief and other minds’ perspective-taking tasks (e.g. Apperly, 2010; Goldman, 2006).

We can now move on to the next variable of our annotation, namely, the source of resonance, which in example (4) was coded as ‘other’, as it originated from the mother, rather than from the child herself. The resonance type in (4) is creative, as the construction includes the addition of the new component 赶紧 *gǎnjǐn* ‘hurriedly’, the omission of the subject 狐狸 *húli* ‘fox’ and the reduction of the verb 逃跑 *táopǎo* ‘run away’. The degree of lexical resonance is 4, as 4 words were repeated from one turn to another: 吓 *xià* ‘be-scared’ 得 *de*, 跑 *pǎo* ‘run’ and 了 *le*. Finally, the degree of syntactic resonance in this case is 5. In fact, what syntactic resonance measures is not the mere repetition of lexical items, but rather the number of internal constituents of a more schematic construct of which both forms are specific instantiations. This corresponds to the comparatively more schematic construction [Subj 吓 *xià* ‘be-scared’ 得 *de* 逃跑 *táopǎo* ‘run’ 了 le]. This partition is illustrated in the diagraph in Table 3.

**Table 3:** Diagraph of the emerging resultative construction [Subj *Xia* De *Taopao* Le] construction

	Subj	*Xia*	De	*Taopao*	Le
MOT	*狐狸*	*吓*	*得*	*逃跑*	*了*
CHI	*/*	*吓*	*得*	(赶紧) *跑*	*了*

We can now look at another example from our dataset to further test our annotation scheme, as given in (5) below:

**Table:** 

(5)		
MOT:	妈妈喜欢这个。	
	māma xǐhuan zhè ge	
	Mom like this CLASS	
	‘Mom likes this one.’	
CHI:	那你玩这个吧!	
	nà nǐ wán zhè ge ba	
	then you play this CLASS BA	
	‘So just play this, come on!’	
		CHILDES / Shanghai / Yezi / 54

In the case above, CHI’s utterance includes the non-obligatory SFP 吧 *ba*, which in Mandarin is used intersubjectively to invite the addressee to agree with a proposition or to engage in a co-action (cf. Tantucci, 2017b, 2021; Tantucci and Wang, 2018, 2020b, 2022). The source of resonance is once again coded as ‘other’, as it originates from MOT. The resonance type is creative, due to the child re-elaborating the priming construction [Subj Tr-Verb^4^*这 zhè 'this'* CLAS^5^] and intervening on the subject, the verb and further adding an intersubjective component, viz. the SFP 吧 *ba* as a surplus of meaning at the end of the sentence. The count of lexical resonance is 2, namely the words 这 *zhè* ‘this’ and 个 *ge*, while syntactic resonance is 4, i.e. the internal constituents of the comparatively more schematic construct [Subj Tr-Verb *这 zhè 'this'* CLAS]. This is illustrated in the diagraph in Table 4.

**Table 4:** Diagraph of the emerging construction [Subj Tr-Verb *this* CLAS]

	Subj	Tr-Verb	*zhè*	CLAS
MOT	*妈妈*	*喜欢*	*这*	*个*
CHI	(那) 你	*玩*	*这*	*个* (吧)

One possible caveat of the present scheme of annotation may regard the identification of schematic structures. Undoubtedly, constructional schematicity pervades dialogic conversation. The identification of schematic constructs informing the dimension of syntactic resonance may therefore represent a challenge for the replicability of the results of the annotation. This issue was tackled by having lexical resonance as a condition for the identification of syntactic resonance. What this means is that at least one priming lexical item had to be among the internal constituents of a ‘syntactically’ resonating construct, e.g. the presence of respectively 这 *zhè* ‘this’ and 个 *ge* in CHI’s turn as necessary conditions for the identification of syntactic resonance for the construct [Subj Tr-Verb 这 *zhè* ‘this’ CLAS] in example (5).

The present framework of analysis was entirely based on formal and replicable criteria of annotation. Along three stages of coding, performed by three different annotators, the Cronbach’s Alpha accuracy of the ‘quantitative’ variation of resonance,, was respectively α = 0.76, α = 0.87 and finally α = 0.92.

## Analysis and Results

One of the most important advances in corpus-based approaches to dialogic syntax is that resonance and overt interactional engagement can be measured both lexically and schematically. Our analysis started by looking at the degree of syntactic resonance in the two populations with a T-test being performed to compare the means of each group.

**Fig. 1:**
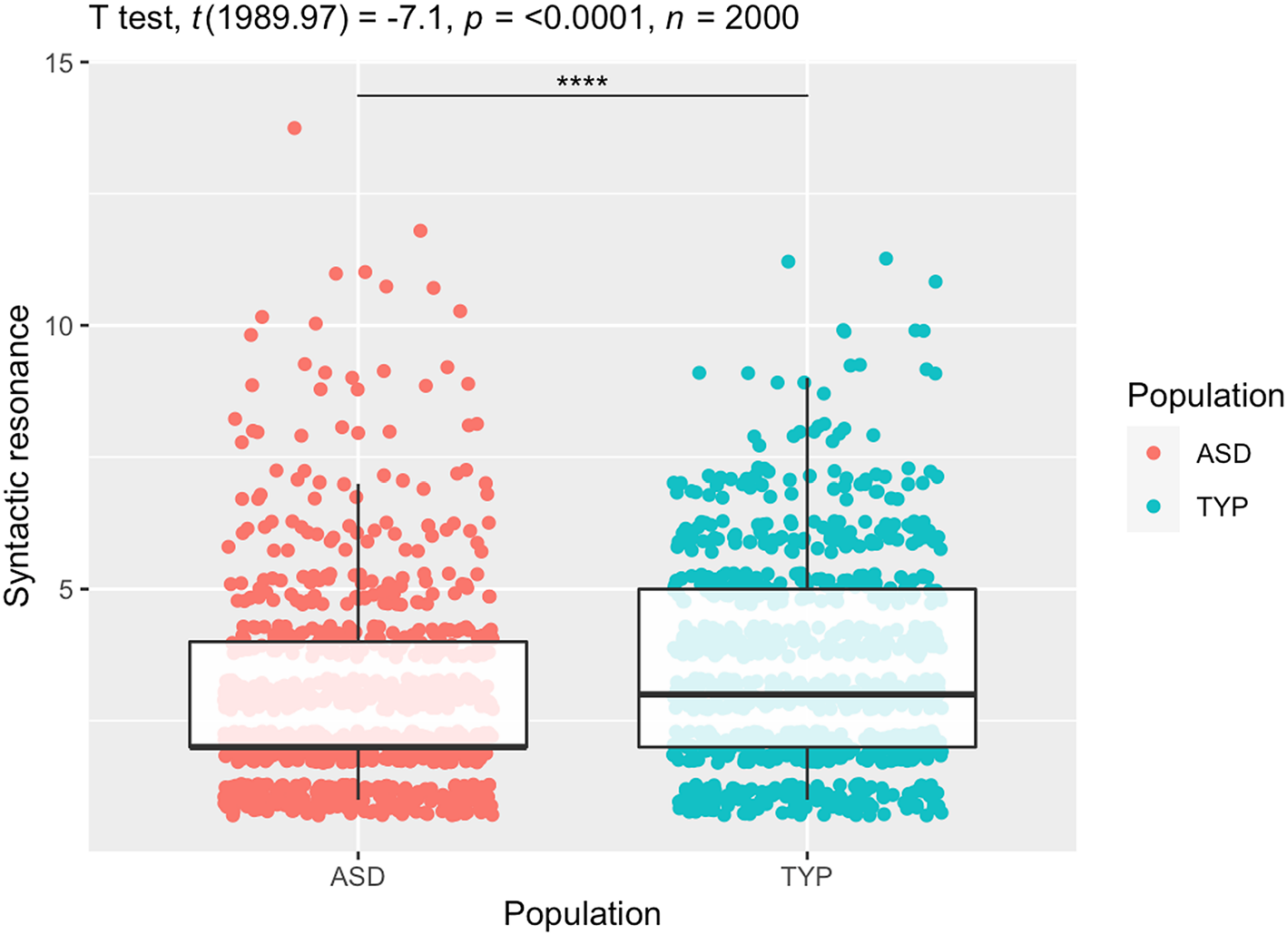
Degree of syntactic resonance in ASD and TYP populations

**Fig. 2:**
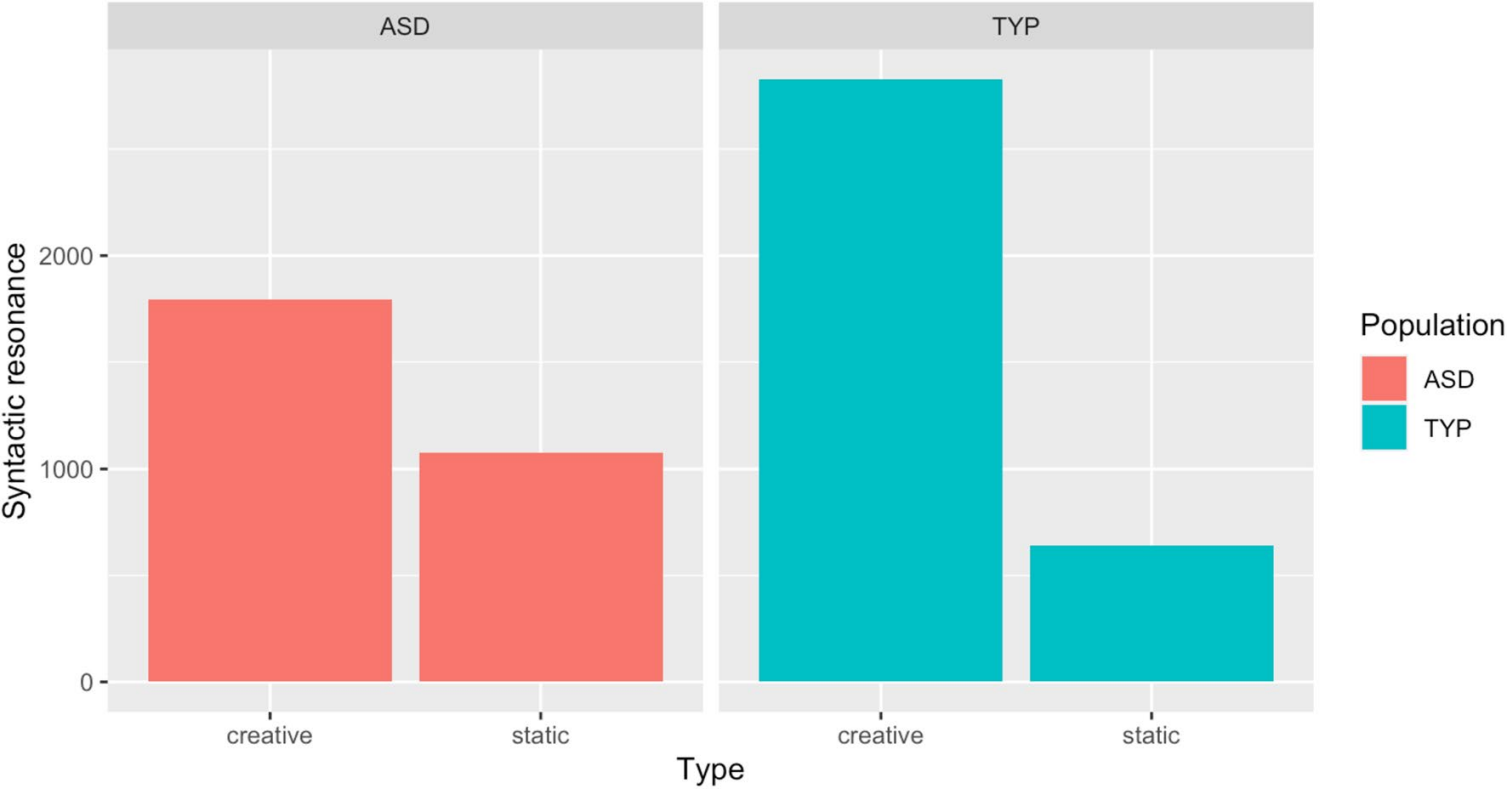
Barplot of creative vs static resonance in ASD vs TYP populations

From the boxplot in Fig. 1, we can clearly see how syntactic resonance as a whole is produced by a significantly larger degree in TYP children (in green) in contrast with children with ASD, in red (*t*(1989) = 7.1, *p* < 0.0001, *n* = 2000). Simply put, when resonance occurs, a significantly higher proportion of schematic linguistic input is re-used, either creatively or statically, by TYP children throughout naturalistic interaction. While this is an important result to report, it is yet not entirely surprising. This indeed demonstrates that resonance as such is less prominent in the ASD population as compared with the TYP one. Nonetheless, what is also remarkable is that such a mismatch appears to be gradient, rather than reflecting an absolutely impeded capacity of children with ASD. This indeed, supports the view that interactional engagement is partly—rather than entirely—impeded in children with ASD, with comparatively weaker, rather than missing propensity to re-elaborate dialogic primes of their interlocutors (e.g. Du Bois, 2014; Hobson et al., 2012; Kissine, 2021). The most obvious research question arising at this point hinges on whether some significant mismatch exists regarding how—rather than how much—ASD children would resonate with a prime in contrast with TYP individuals. The degree of syntactic resonance occurring creatively vs statically in the two populations is reported in the barplot from Fig. 2.

From the above, we can clearly see how resonance tends to occur creatively to a larger extent in the typically developing (TYP) population as opposed to the ASD one. Even more crucially, it appears that values of static resonance are, in turn, comparatively higher in the ASD group in contrast with the TYP one. This suggests a more impeded capacity to engage creatively with a prime in children with ASD. That is, ASD children do engage with dialogic primes, however this may more distinctively involve an ego-centric learning process, as opposed to the ability to provide a new contribution to the here-and-now of the interaction. We thus fitted a mixed effects linear regression model (cf. Baayen et al., 2008) as our goal was now predicting the degree and the modality in which children would resonate across the two populations. As we were interested in the relationship between creativity and engagement, we controlled for overt intersubjectivity at sentence periphery by including all utterances that comprised non-obligatory sentence final particles of intersubjective engagement (SFP). Finally, we fitted the children’s names as a random variable. The results of our model are reported in Table 5.

**Table 5:** Mixed effects linear regression of Resonance type in contexts of explicit engagement

Random effects				
Groups	Name	Variance	Std. Deviation	
Name	(Intercept)	.0.109	0.33	

Fixed effects				
	Estimate	Std. Error	T value	Pr( >|t|)
(Intercept)	2.4450	0.1551	15.769	1.04e−14***
Population TYP	− 0.2035	0.2050	−0 .993	0.327
Creative	0.9090	0.1153	7.885	5.16e−15***
Population TYP:Creative	0.7846	0.1681	4.667	3.26e−06***

**Fig. 3:**
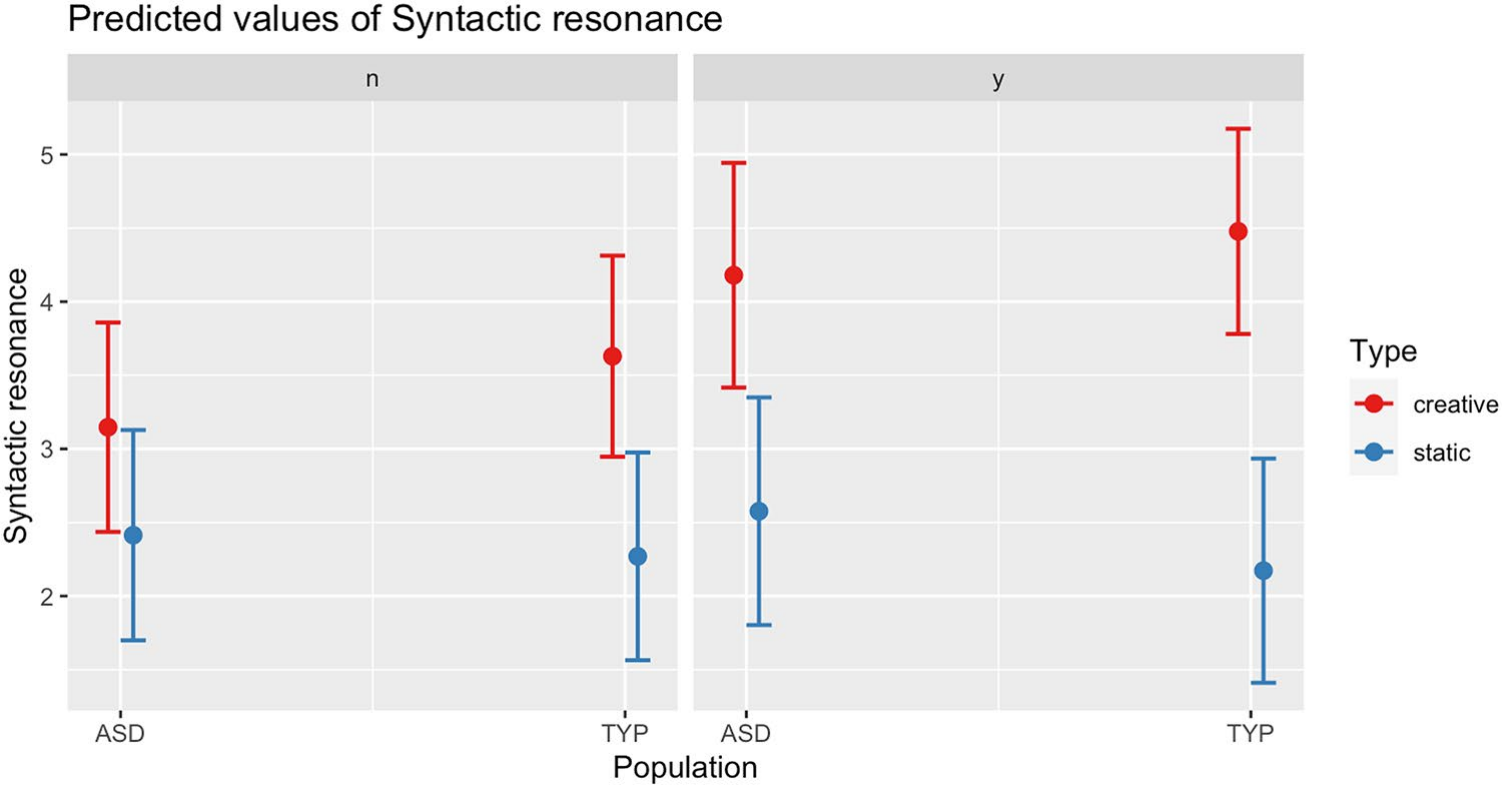
Predicted values of syntactic resonance across TYP vs ASD populations

At the top of Table 5, are reported the random effects of the model, including the standard deviation, which shows the variability from the predicted values, with reference to the names of the children of both corpora. The fixed effects appear at the lower part of the table. Here, the Estimate column indicates the coefficients of the slope for the fixed effects on the degree of syntactic resonance, i.e. TYP vs ASD population and Creative vs Static resonance. From the above, we can first see that the coefficient for syntactic resonance is positive in combination with creative re-elaboration of a dialogic prime, in contrast with static resonance (Creative, *β*(1970) = 0.909, p < 0.005). This indicates that in contexts of explicitly marked intersubjective engagement, resonance shows a significant tendency to occur as a creative phenomenon, underpinning the addition of new morphosyntactic and pragmatic information resulting from a dialogic input. This is a fundamental result, as it indicates that marked intersubjective engagement (controlled via presence of SFP) is a productive environment of interactional creativity. While this phenomenon emerged to be significant across both populations, it is yet at play to a significantly larger extent in the typically developing population as opposed to the ASD one (TYP:Creative, *β*(1991) = 0.784, p < 0.005). In Fig. 3 below are plotted the coefficents of predicted syntactic resonance in presence vs absence of sentence final particles of overt intersubjective engagement in static vs creative resonance conditions across the two populations.

With reference to the plot in Fig. 3, a number of important observations are in order. First, as shown in the left quadrant, the predicted values of Syntactic resonance occurring statically and without overt particles of intersubjective engagement (the quadrant is accordingly labelled as ‘n’) is very close across the two populations (both around 2.5). What this means is that automatic imitation of a dialogic stimulus—presumably occurring mostly as a learning process—is not distinctively inhibited in ASD children, who actually display a marginally higher coefficient under such conditions. A second fundamental element of the plot is that the blue coefficients for creative resonance are higher across both populations and in all conditions. This indicates that a higher portion of schematic information is processed by children when creativity is at play, entailing a stronger linguistic engagement with a peer (i.e. more language is produced in return to the original prime). This point is particularly important as it indicates that creativity correlates with heavier linguistic processing and stronger interactional engagement. A third important insight to be drawn for Fig. 3 is that values for creative resonance are comparatively higher in the TYP population, most remarkably so in conditions of overt engagement with a peer via sentence final particles of intersubjectivity (SFP), as shown in the right quadrant labelled as ‘y’. Here, the mismatch between creative and static resonance is extremely evident with TYP children, suggesting that when overt intersubjective engagement via SFP is at play, resonance distinctively occurs as a creative phenomenon. While this mismatch is also present in ASD children, however the gap between static and creative resonance is much less prominent. The key here is that while creative resonance in children with ASD indeed increases with presence of sentence final particles of intersubjective engagement (SFP), however the latter is not as decisive as a condition for creative instead of static re-elaboration of a prime. In fact, when SFP are in action, even static resonance tends to marginally grow in children with ASD, in contrast with the TYP population. This mismatch can be captured with a conditional inference tree model, along with the source of resonance (cf. Hothorn et al., 2006; Tagliamonte & Baayen, 2012).

**Fig. 4:**
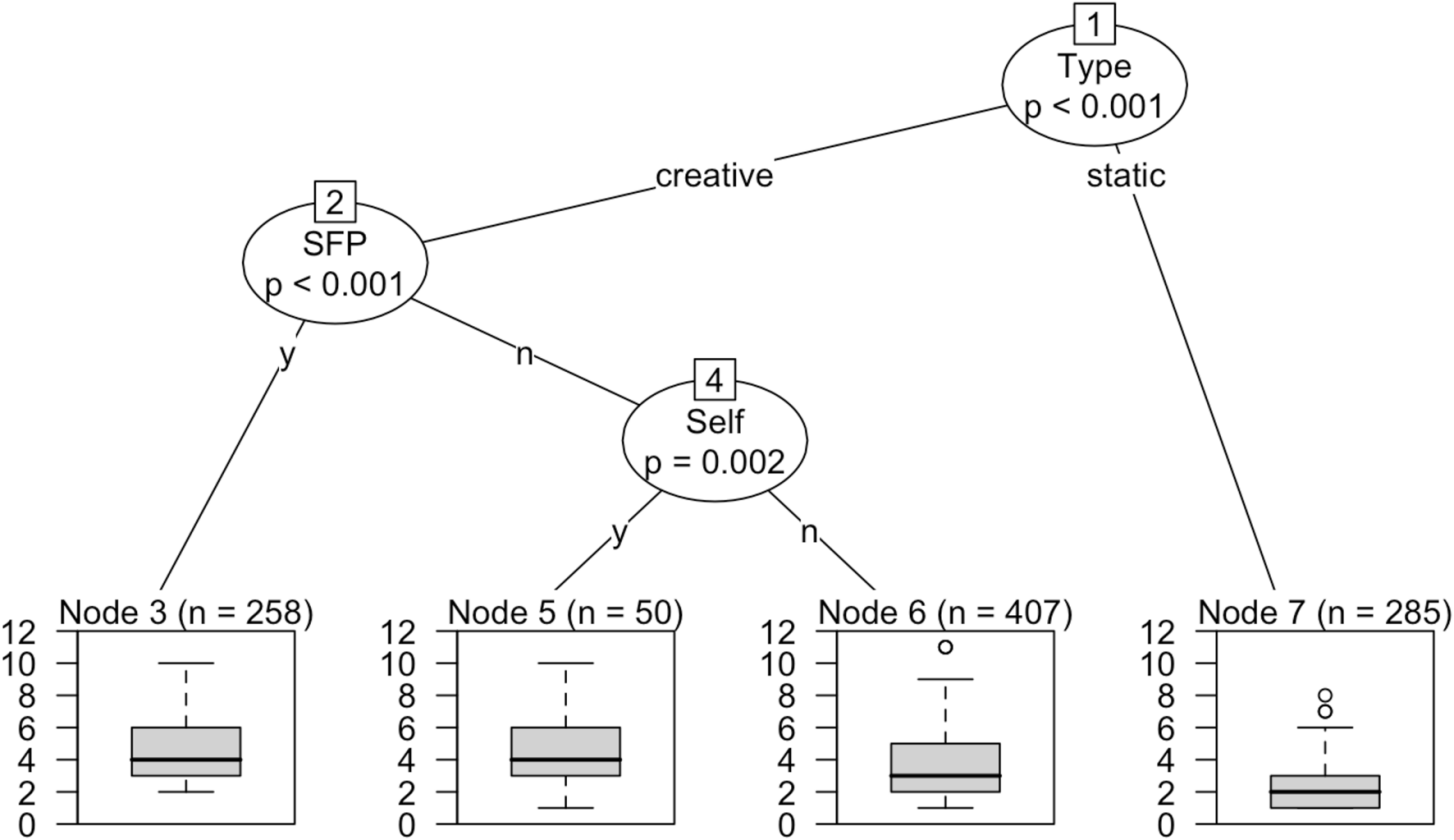
Conditional inference tree for the prediction of syntactic resonance in the ASD population

**Fig. 5:**
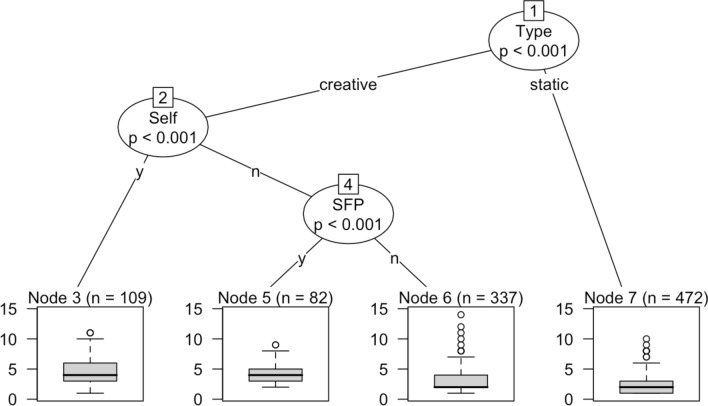
Conditional inference tree for the prediction of syntactic resonance in the ASD population

The models in Figs. 4 and 5 are fitted with the ‘ctree’ function of the R package ‘party’ (cf. Levshina, 2015, p. 291). The conditional dependencies among variables are ranked in a descending order and are based on statistical significance (the higher the node, the more significant the partition of each split). At each node, a conditional decision among the predictors is computed in order to assess the weight of syntactic resonance in either the ASD or the TYP population. At the bottom of each model are reported the boxplots of syntactic resonance that result from each decisional path. One way to look at the models could be in terms of a computational re-enactment of a number of conditional decisions that statistically determine the degree of syntactic resonance in children’s response to a dialogic stimulus. We can clearly see how resonance occurring statically triggers the lowest levels of linguistic information in both populations (see the link between node 1 and node 7 at the right hand-side of each plot). This further supports the conclusion that lack of creativity results in lower degree of linguistic engagement. Even more importantly, the two plots show an opposite ranking under creative conditions. On the one hand, in Fig. 4 TYP children most significantly rely on the employment of SFP (as overt markers of intersubjective engagement) in order to process highest levels of dynamic resonance (see nodes 2 to 3). On the other hand, what is statistically most crucial for the degree of syntactic resonance in the ASD population is whether the child creatively resonates with him/herself or with the mother. As shown in Fig. 5, values of ASD creative resonance are indeed higher when the child egocentrically resonates with what s/he just said (see the relationship between node 2 and 3). What this ultimately indicates is that creative resonance is primarily intersubjective in the case of TYP children, being most strongly associated with overt engagement via SFP. Quite differently, ASD children most significantly resonate ego-centrically, viz. when they are themselves the source of their resonating construction.

## Discussion

Creativity is key for interactional engagement. As insightfully put in Hurley (2008, p. 4), a fundamental difference between copying ends and copying means is at play for theorising the phylogeny of enactive imitation and action understanding. What she calls ‘true imitation’ involves the re-calibration of a given action for different ends and a given end pursued by various means (Barkley, 2001, p. 8; Tomasello, 1999). This is something humans are distinctively good at, while it is rare to find evidence of true re-enactive imitation of this kind in nonhuman animals (Byrne, 1995; Tomasello, 1996; Voelkl & Huber, 2000; Zentall, 2001). Creative resonance underpins the re-combinant re-enactment of a prime with the goal of expressing something new. Large-scale data from the present study shed light on the way children with ASD tend to engage both statically and creatively with a stimulus in a somewhat different way than TYP children. Firstly, they display less linguistic engagement as a whole, no matter how resonance is achieved interactionally. Secondly, they also show a relatively impeded ability to creatively recombine a preceding dialogic prime in comparison with the typically developed population. Most crucially, they showed a relatively inhibited capacity to engage creatively with linguistic primes in combination with overt sentence final particles of intersubjectivity (SFP). The latter are a non-obligatory grammaticalised category in Mandarin Chinese (and a number of languages of the South East) and constitute a fundamental diagnostic for assessing whether a speaker purposely makes overt his/her concern for the potential reactions of an on-going utterance (Tantucci, 2021). In fact, when creativity is at play, ASD children show a remarkable preference for self-engagement, as they significantly tend to primarily resonate with themselves, rather than with their own interlocutors. The results of our study support the gradient stance towards impeded engagement in ASD that is proposed in Du Bois et al. (2014) and Hobson et al. (2012). However, it also provides new compelling insights on the relationship between creativity and engagement in naturalistic interaction.

In the heated debate about the nature of mindreading and intersubjectivity not much emphasis has yet been placed on the non-propositional nature of human interaction, hinging on degrees of engagement that interactants and social members require for successful cooperation (cf. Tantucci, 2021). In this sense, the present model provides a fine-grained framework for assessing the degree to which interlocutors are able to linguistically display interactional engagement with a peer. More importantly, what emerged from this study is that engagement correlates with creativity (Tantucci, Culpeper and Di Cristofaro, 2018; Culpeper and Tantucci, 2021), as it involves the ability to re-combine the meaning and the structure of a peer’s preceding utterance. However, in the ASD population, engagement is not as a strong predictor of creativity as it is with TYP children. In fact, engagement in ASD speech is somewhat preserved at the expense of creativity, and the other way around. Creative re-elaboration of interactional primes originating from other speakers exists in ASD speech, yet not as an inherent byproduct of engagement. This may suggest that overtly engaging interaction is a matter of an explicit choice in the ASD population, whereby executive functioning resources are to be either allocated to recombinant and novel alterations of a prime or, rather, to overtly marked engagement with an interlocutor. An important question regarding the relationship between language production and mindreading abilities is considered by Kissine (2021). Namely, if mindreading is necessary for the development of linguistic skills and competence—as it is assumed in most constructionist approaches (e.g. Green et al. 2010; Tomasello, 2003)—then what is the explanation for linguistic skills that are nonetheless acquired and developed across the autistic spectrum? Kissine notes that differences rather than deficiencies are at play regarding the ‘modality’ in which interactional abilities are acquired in ASD populations. The present paper provided evidence supporting this assumption, as interactional engagement in ASD children is not inherently inhibited, but rather as ‘one possible choice’ of constructional organisation, rather than a necessary motivation. This may indicate that what is inhibited in ASD individuals is the convergence between engagement and creativity. What our data ultimately shows is that both components are indeed present in ASD speech, yet without converging to the same degree as they do in TYP interaction.

## Limitations

The present study has some limitations. While on the one hand the analysis relies on large scale data from naturalistic interaction, multimodal components of resonance, involving prosody, intonation and gestures were not part of the dataset at our disposal. In this sense, we believe that future research adopting a similar methodology would greatly benefit from extra-linguistic components that may themselves contribute to resonance and shed new light on interactional creativity and engagement. One second limitation has to do with the fact that only one language has been tackled in the present account. While mismatches involving intersubjectivity at sentence periphery have already been attested cross-linguistically (e.g. Tantucci & Wang, 2018, 2020a, b), it would be necessary to put the present model of analysis into play for other languages as well, especially ones that do not include a grammaticalised system of sentence final particles of intersubjectivity.

## Conclusions

This paper provided a novel applied method to empirically measure morphosyntactic creativity and engagement in naturalistic interaction. In this specific case, it tackled a number of important questions in research on priming and complex imitation in neurotypical and ASD children’s speech. Firstly, it provided large-scale corpus-based data showing that interactional engagement with a dialogic prime correlates significantly with creative resonance. Our data indicate that this tendency underpins both neurotypical and ASD speech, despite being less prominent in the latter population. Children with ASD showed a preference to engage creatively with their own linguistic turns at talk (in the form of self-priming) when resonance was at play. On the other hand, they overtly marked their engagement with a peer via sentence final particles (SFP) of intersubjectivity at the expense of creative recombination of a previous utterance. This was in sharp contrast with what neurotypical children did. In this latter case, overtly marked intersubjective engagement via SFP significantly occurred as a byproduct of creative recombination of previous turns at talk. What these results indicate is that children with ASD indeed seem to acquire both abilities to spontaneously express overtly marked interactional engagement and to creatively intervene on previously encountered dialogic constructions. However, when compared with the neurotypical population, they show a tendency to either favour one mechanism or the other. This suggests a competition among explicit engagement and creative language production, presumably as a partition of executive functioning during the here-and-now of the conversation. Put simply, children with ASD struggle more than neurotypical children in allocating cognitive resources to engagement and interactional creativity at the same time. This, in turn, supports the view that intersubjective engagement—and potentially mindreading—is not inherently inhibited in ASD, but rather functions as one ‘possible mechanism of conceptualisation’, which requires ad hoc conceptual focus and effort. This is in contrast with the view that intersubjective engagement constitutes than an absolute pre-condition for linguistic proficiency and an inherent component of interactional abilities.

## Footnotes

1 Priming is here to be intended as a conceptual mechanism whereby exposure to one stimulus influences a response to a subsequent stimulus.

2 The dataset that we used did not include specific diagnostic information about every individual child.

3 Intensifier.

4 Transitive verb.

5 Classifier.
