# Correction to: Dialogic Priming and Dynamic Resonance in Autism: Creativity Competing with Engagement in Chinese Children with ASD

**DOI:** 10.1007/s10803-022-05594-z

**Published:** 2022-05-09

**Authors:** Vittorio Tantucci, Aiqing Wang

**Affiliations:** 1 Department of Linguistics and English Language Lancaster University, Lancaster, UK; 2 Chinese Department of Modern Languages and Cultures, University of Liverpool, Liverpool, UK

## Correction to: Journal of Autism and Developmental Disorders 10.1007/s10803-022-05505-2

In the original article there is an error in reference section.

(e.g. a number of Du Bois 2014 citations should actually be Du Bois et al. 2014).

This has been corrected in this erratum.

The original article has been corrected

